# Synthesis of ω-Methylsulfinyl- and ω-Methylsulfonylalkyl Glucosinolates

**DOI:** 10.3390/molecules30030704

**Published:** 2025-02-05

**Authors:** Manolis Mavratzotis, Stéphanie Cassel, Gina Rosalinda De Nicola, Sabine Montaut, Patrick Rollin

**Affiliations:** 1 ICOA, CNRS, Université d’Orléans, UMR 7311, BP 6759, F-45067 Orléans, Francestephanie.cassel@univ-tlse3.fr (S.C.); patrick.rollin@univ-orleans.fr (P.R.); 2 Laboratoire SOFTMAT, UMR CNRS 5623, Université P. Sabatier Toulouse III, 118 Route de Narbonne, 31062 Toulouse CEDEX 9, France; 3 Research Centre for Vegetables and Ornamental Crops, Council for Agricultural Research and Economics (CREA), Via dei Fiori 8, 51017 Pescia, Italy; 4 School of Natural Sciences, Laurentian University, 935 Ramsey Lake Road, Sudbury, ON P3E 2C6, Canada

**Keywords:** glucosinolates, thiohydroximates, sulfoxides, sulfones

## Abstract

General pathways were devised to synthesize ω-methylsulfinyl- and ω-methylsulfonylalkyl glucosinolates, which represent an important class of structurally homogeneous plant specialized metabolites. The first approach was based on the selective *S*-oxidation of ω-methylsulfanyl analogs previously obtained in our laboratory, producing the corresponding sulfoxide or sulfone counterparts in moderate yields. In an alternative approach, previously prepared ω-nitroalkyl methylsulfide precursors were selectively oxidized either to sulfoxides or to sulfones. The key-thiofunctionalized hydroximoyl chloride intermediates were prepared in situ from sulfoxides or sulfones using a nitronate chlorination strategy. A coupling reaction with 1-thio-β-d-glucopyranose was directly applied, followed by *O*-sulfation of the intermediate thiohydroximates. The final deprotection of the sugar moiety produced the target compounds, including renowned glucoraphanin and homologs, intended for further bioactivity investigations.

## 1. Introduction

**Figure 1:**
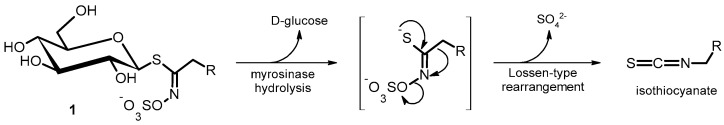
Glucosinolate (GSL) general structure **1** and their myrosinase-catalyzed hydrolysis to isothiocyanates (ITCs).

Glucosinolates (GSLs) **1** are thiosaccharidic specialized metabolites almost exclusively found in all families of the plant order Brassicales. All of the ca. 140 known GSLs display a remarkable structural homogeneity based on a *β*-d-glucopyrano unit, bearing an *O*-sulfated anomeric (*Z*)-thiohydroximate function connected to an aglycon R, the constitution of which is the sole structural variant, depending on plant species. Associated with the enzyme myrosinase (E.C. 3.2.1.147), GSLs can undergo hydrolytic cleavage of the anomeric C–S bond with d-glucose release. The liberated aglycon ordinarily undergoes a Lossen-type rearrangement to mainly produce isothiocyanates (ITCs) (Figure 1) [1,2,3].

**Figure 2:**
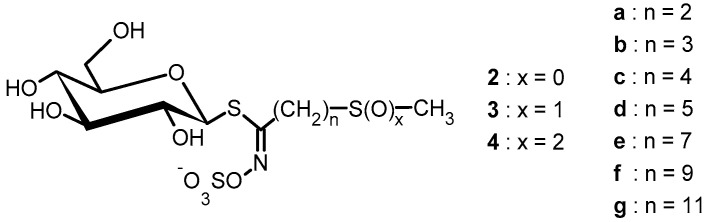
The general structure of glucosinolates (GSLs) bearing a ω-thiofunctionalized alkyl chain. Legend used for GSLs mentioned in this study: **2a**–**g**, ω-methylsulfanyl-; **3a**–**g**, ω-methylsulfinyl-; **4a**–**g**, ω-methylsulfonylalkyl GSL.

Most strikingly, over a third of the actually registered GSL structures contain a terminal thio-function—namely sulfide (**2**), sulfoxide (**3**), or sulfone (**4**)—attached to their aglycon alkyl chain, as shown in Figure 2 [4,5].

Within the order Brassicales, GSLs bearing a ω-thiofunctionalized alkyl chain are mainly found in Brassicaceae and Capparaceae, albeit also frequently as minor components in the GSL profile of other families. However, their diversified bioactivity potential has long been recognized over a broad range of aglycon chain lengths (Figure 2). To highlight one most bio-relevant example, 4*R_S_*-(methylsulfinyl)butyl GSL (glucoraphanin, **3c**) [6,7,8], bearing a 4*R_S_*-methylsulfinylbutyl aglycon stands as a popular source of bioactive sulforaphane (4*R_S_*-(methylsulfinyl)butyl ITC) [9,10,11]. Noteworthy, recent findings are shedding light on the traditionally accepted view that the activity of GSLs is exclusively mediated by their degradation products: ITCs. A powdered extract obtained from broccoli seed indicated **3c** as a potential agent effective in preventing obesity and related metabolic disorders in high-fat diet (HFD)-fed mice [6]. In recent work, Lucarini et al. found that **3c** releases hydrogen sulfide (H_2_S) in a cysteine-dependent fashion similar to its ITC sulforaphane, suggesting that **3c** may be endowed with biological activity based on its H_2_S-releasing properties [12]. The investigation of direct bioactivities of intact GLSs, particularly ω-thiofunctionalized GSLs, is steadily increasing. Pure **3c** obtained from Tuscan black kale seed, according to an established method [13], reduced neuropathic pain in different animal models [12] and improved behavior in rats that were associated with depressive-like phenotypes [14]. Moreover, **3c** supplementation was suggested as a useful strategy for prevention and treatment of sarcopenia [15].

Accessing naturally occurring GSLs from plants through extraction processes is generally not straightforward [16,17]. Vegetable sources, which can be considered optimal for an efficient purification of one specific GSL in a reasonable amount, are in very limited numbers [18,19,20]. In fact, plant materials suitable for this purpose have to meet two important characteristics, namely a profile with a restricted number of GSLs and a high content of the GSL of interest [13,21]. Therefore, the synthetic approach could represent a valid alternative to making GSLs available as attractive substrates for biological studies [22].

Nevertheless, since our pioneering study [23], only scarce synthetic efforts have focused on GSLs bearing an external thio-function in the aglycon part. With the aim of analytical and metabolic studies, Botting et al. synthesized isotopically labeled glucoraphanin **3c** [24,25] and 4-(methylsulfanyl)butyl GSL (glucoerucin, **2c**) [26]. Some additional synthetic achievements have also been reported [27,28]. In the continuation of our previous work [29], we report here general synthetic pathways intending to deliver ω-methylsulfinyl- and ω-methylsulfonylalkyl GSLs **3a**–**g** and **4a**–**g** (Figure 2) for further bioactivity evaluations.

## 2. Results and Discussion

### 2.1. Synthetic Pathway 1

**Scheme 1:**
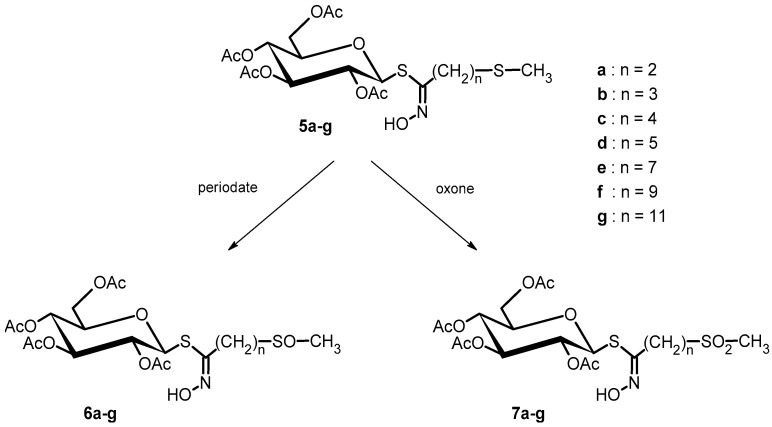
Pathway 1—oxidation of ω-methylsulfanylalkyl thiohydroximate precursors **5a**–**g** [29] to yield protected glucosyl ω-methylsulfinyl and ω-methylsulfonylalkyl counterparts **6a**–**g** and **7a**–**g**, using the periodate and oxone processes, respectively.

Based on our previous investigations of GSL oxidation [30], a first option could be envisaged to access GSL sulfoxides and GSL sulfones. This would implement a strategy of controlled oxidation of the protected glucosyl ω-methylsulfanyl intermediates **5**, previously synthesized in our laboratory, to obtain GSL sulfides **2** [29] (Scheme 1).

We were aware of the randomness of this process by relying on preliminary oxidation tests applied to model thiohydroximates, which confirmed the appreciable sensitivity of the anomeric sulfur in oxidative media. In this way, the C1-S bond is cleaved, resulting in glucose expulsion. However, when applying carefully controlled conditions in both periodate and oxone processes, we were pleased to observe a satisfactory outcome. Thus, our sulfide precursors **5a**–**g** could be smoothly converted into the corresponding sulfoxides **6a**–**g** in 42 to 68% yields by the periodate process. A similar conversion could be carried out with oxone, producing the corresponding sulfones **7a**–**g** in yields ranging from 49 to 70%. It is noteworthy that in both reactions, the highest yields were obtained for the thiohydroximates bearing the longest aglycon chains (Table 1). The overall assessment for the conversion of the sulfide precursors **5** was a yield range of 13 to 36% for ω-methylsulfinyl thiohydroximates **6** and 16 to 39% for ω-methylsulfonyl thiohydroximates **7**, depending on the length of the aglycon chain, as summarized in Table 1.

**Scheme 2:**
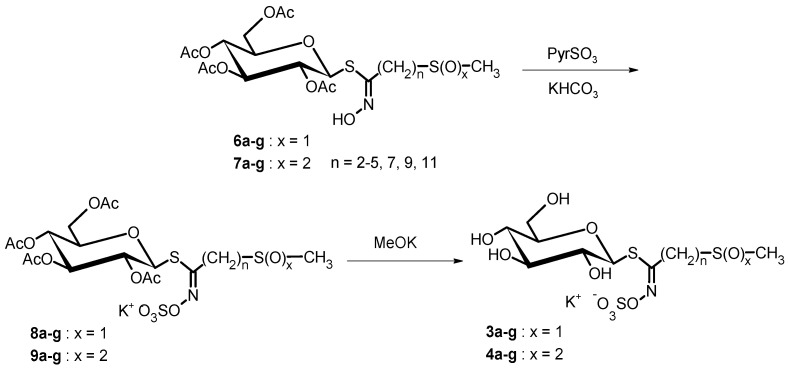
General conversion of thiohydroximates **6a**–**g** and **7a**–**g** into per-*O*-acetylated glucosinolates (GSLs) **8a**–**g** and **9a**–**g** and final deprotection of the glucopyranosyl moiety to produce GSLs **3a**–**g** and **4a**–**g**.

The final conversion of the thiohydroximates **6a**–**g** or **7a**–**g** into the target ω-methylsulfinyl GSLs **3a**–**g** or ω-methylsulfonyl GSLs **4a**–**g** was performed according to a previously described protocol [29]. *O*-sulfation of the hydroximino moiety using sulfur trioxide pyridine complex (PyrSO_3_), followed by quenching with aqueous potassium hydrogen carbonate, produced the per-*O*-acetylated GSLs **8a**–**g** (yield range 54–78%) and **9a**–**g** (yield range 56–85%). The carbohydrate moiety was finally deprotected by base-catalyzed methanolysis to produce the corresponding GSLs **3a**–**g** (yield range 80–93%) and **4** (yield range 80–95%) (Scheme 2).

### 2.2. Synthetic Pathway 2

**Figure 3:**
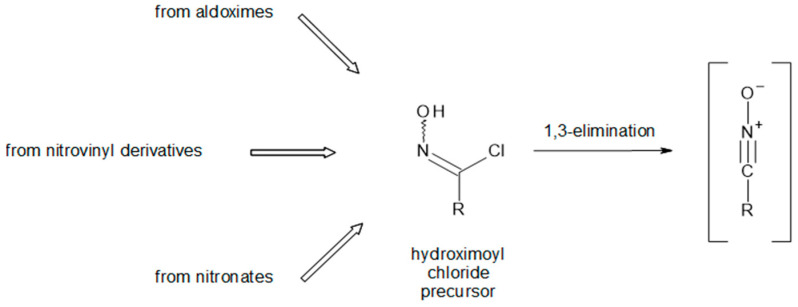
Methods to generate nitrile oxides.

Having developed in previous work [29] straightforward synthetic processes for the preparation of ω-methylsulfanylnitroalkanes, it was decided to build up an alternative approach to the target thiofunctionalized GSLs **3** and **4**. Most synthetic methods developed for GSLs over the years involve as a key step the *Z*-stereospecific formation of a thiohydroximate function, usually resulting from the 1,3-addition of a glucosyl mercaptan onto a transient nitrile oxide [22]. As they are generally unstable molecular species, nitrile oxides have to be generated in situ through the base-induced conversion of hydroximoyl chlorides, which in turn currently result from the chlorination of aldoximes or nitronates (Figure 3).

**Scheme 3:**
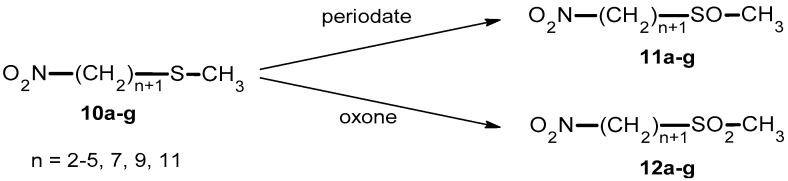
Pathway 2a—oxidation of sulfide precursors **10a**–**g** [29] to sulfoxides **11a**–**g** or sulfones **12a**–**g**.

Taking advantage of the readily available ω-nitroalkyl methylsulfide precursors **10a**–**g** described in our previous work [29], chemoselective oxidation was applied to obtain sulfoxide homologs **11a**–**g** and sulfone homologs **12a**–**g**, using either sodium periodate or oxone, respectively (Scheme 3).

**Scheme 4:**
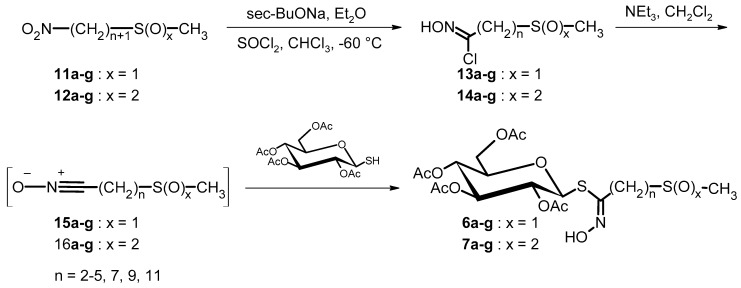
Pathway 2b—synthetic sequence involving in situ generation of intermediate nitrile oxides from hydroximoyl chlorides **13a**–**g** or **14a**–**g**, followed by peracetylated 1-thio-glucopyranose coupling to deliver glycosyl thiohydroximates **6a**–**g** and **7a**–**g**.

Following a one-pot nitronate chlorination strategy initially established by Kjaer [31], all nitro-compounds **11a**–**g** or **12a**–**g** were converted into the corresponding hydroximoyl chlorides **13a**–**g** and **14a**–**g** through a two-step process. Nitroalkanes were first treated with sodium sec-butoxide and then chlorinated in chloroform with thionyl chloride at low temperature (−60 °C). The hydroximoyl chlorides were used without further purification by base-catalyzed (NEt_3_) 1,3-elimination. In situ generated intermediate nitrile oxides **15a**–**g** and **16a**–**g** were used as electrophilic acceptors for coupling with 2,3,4,6-tetra-*O*-acetyl-1-thio-ω-d-glucopyranose to give the protected thiohydroximate **6a**–**g** and **7a**–**g** [32] (Scheme 4).

This stereospecific addition step produced the corresponding ω-methylsulfinylalkyl- and ω-methylsulfonylalkyl (*Z*)-thiohydroximates in yields ranging from 30 to 54% for sulfoxides **6a**–**g** and 35 to 55% for sulfones **7a**–**g** (Table 2). Similarly to results obtained along pathway 1, the highest yields were obtained for the thiohydroximates bearing the longest aglycon chains (Table 2). The overall assessment for the conversion of the sulfide precursors **10** was a yield range of 18 to 51% for ω-methylsulfinyl thiohydroximates **6**, and 25 to 52% for ω-methylsulfonyl thiohydroximates **7**, depending on the length of the aglycon chain, as reported in Table 2.

The final conversion of the thiohydroximates **6a**–**g** or **7a**–**g** into the target ω-methylsulfinyl GSLs **3a**–**g** or ω-methylsulfonyl GSLs **4a**–**g** was performed as reported above, according to Scheme 2 through the *NO*-sulfation of the glucosyl thiohydroximate, followed by deprotection of the glucopyranosyl moiety.

To sum up, seven ω-methylsulfinyl- and seven ω-methylsulfonylalkyl GSLs were produced and fully characterized. GSLs **3a**–**g** and **4a**–**g** were obtained with an overall yield of 6–25% and 7–31% through pathway 1. Conversely, pathway 2 produced the desired compounds with an overall yield of 13–39% and 16–43%, respectively.

## 3. Experimental Section

### 3.1. General Information

All chemicals were purchased from Sigma-Aldrich and used without further purification unless otherwise stated. The details of the general information referring to procedures and instruments used for the purification and full characterization of compounds are the same as those previously reported [29].

### 3.2. Organic Synthesis

#### 3.2.1. Pathway 1

##### Selective Oxidation of ω-Sulfides **5** to ω-Sulfoxides **6**

Glucosyl thiohydroximates **5a**–**g** were obtained as previously described in [29]. To a stirred solution of the protected glucosyl, thiohydroximate **5** (1 mmol) in methanol (30 mL) cooled at 0 °C and an ice-cold solution of NaIO_4_ (2 mmol) in water (10 mL) was added dropwise. After stirring at r.t. for 1.5–3 h, the mixture was filtered, and the clear solution was diluted with water (50 mL) and then repeatedly extracted with chloroform (4 × 30 mL). The combined organic phases were dried over Na_2_SO_4_, filtered, and concentrated in vacuo. The residue was purified by flash column chromatography (dichloromethane containing 4–6% *v*/*v* methanol) to produce sulfoxides **6a**–**g** as amorphous solids.

*S-(2,3,4,6-Tetra-O-acetyl-β-d-glucopyranosyl) (Z)-3-(methylsulfinyl)propanethiohydroximate* *(***6a***).*

White amorphous powder (0.21 g, 42% yield), [α]_D_ -16 (*c* = 0.6, CHCl_3_). ^1^H NMR (CDCl_3_): δ 1.98, 2.01, 2.02, 2.05 (4s, 12H, *Me*CO), 2.62 (s, 3H, *Me*SO), 2.85–2.98 (m, 2H, H-8), 3.00–3.14 (m, 2H, H-9), 3.83 (ddd, 1H, *J*_4,5_ = 10.2, H-5), 4.11 (dd, 1H, *J*_5,6b_ = 2.6, *J*_6a,6b_ = 11.0, H-6b), 4.21 (dd, 1H, *J*_5,6a_ = 5.1, H-6a), 4.95–5.13 (m, 3H, H-4, H-1, H-2), 5.24 (ft, 1H, *J*_3,4_ = 9.0, H-3), 8.54 (bs, 1H, NOH). ^13^C NMR (CDCl_3_): δ 20.1, 20.2, 20.3 (*Me*CO), 24.9 (C-8), 37.7 (*Me*SO), 50.3 (C-9), 61.6 (C-6), 67.7 (C-4), 69.9 (C2), 73.6 (C-3), 75.3 (C-5), 79.2 (C-1), 147.6 (C=N), 169.6, 169.7, 170.3, 171.0 (C=O). HR-ESI-MS *m*/*z* calcd for C_18_H_27_NO_11_S_2_ 497.1026, found 497.1011.

*S-(2,3,4,6-Tetra-O-acetyl-β-d-glucopyranosyl) (Z)-4-(methylsulfinyl)butanethiohydroximate* *(***6b***).*

White amorphous powder (0.25 g, 49% yield), [α]_D_ -17 (*c* = 0.9, CHCl_3_). ^1^H NMR (CDCl_3_): δ 1.97, 2.00, 2.02, 2.05 (4s, 12H, MeCO), 2.08–2.19 (m, 2H, H-9), 2.60 (s, 3H, *Me*SO), 2.67–2.86 (m, 4H, H-8, H-10), 3.83 (ddd, 1H, *J*_4,5_ = 10.3, H-5), 4.12 (dd, 1H, *J*_5,6b_ = 2.6, *J*_6a,6b_ = 11.1, H-6b), 4.19 (dd, 1H, *J*_5,6a_ = 5.4, H-6a), 4.96–5.14 (m, 3H, H-4, H-1, H-2), 5.25 (ft, 1H, *J*_3,4_ = 9.4, H-3), 10.0 (bs, 1H, NOH). ^13^C NMR (CDCl_3_): δ 20.4, 20.5, 20.6 (MeCO), 20.2 (C-9), 31.1 (C-8), 38.2 (*Me*SO), 52.7 (C-10), 61.9 (C-6), 67.9 (C-4), 70.1 (C-2), 73.7 (C-3), 75.7 (C-5), 79.7 (C-1), 147.2 (C=N), 169.4, 169.6, 170.2, 170.7 (C=O). HR-ESI-MS *m*/*z* calcd for C_19_H_29_NO_11_S_2_ 511.1182, found 511.1177.

*S-(2,3,4,6-Tetra-O-acetyl-β-d-glucopyranosyl) (Z)-5-(methylsulfinyl)pentanethiohydroximate* *(***6c***) [28].*

White amorphous powder (0.28 g, 53% yield), [α]_D_ -16 (*c* = 0.6, CHCl_3_). ^1^H NMR (CDCl_3_): δ 1.77–1.91 (m, 4H, H-9, H-10), 1.99, 2.01, 2.03, 2.06 (4s, 12H, *Me*CO), 2.47–2.55 (m, 2H, H-8), 2.59 (s, 3H, *Me*SO), 2.68–2.85 (m, 2H, H-11), 3.75 (ddd, 1H, *J*_4,5_ = 9.8, H-5), 4.12 (dd, 1H, *J*_5,6b_ = 2.6, *J*_6a,6b_ = 12.5, H-6b), 4.18 (dd, 1H, *J*_5,6a_ = 5.1, H-6a), 4.98–5.11 (m, 3H, H-4, H-1, H-2), 5.25 (dd, 1H, *J*_3,4_ = 9.0, H-3), 7.87 (bs, 1H, NOH). ^13^C NMR (CDCl_3_): δ 20.4, 20.5, 20.6 (*Me*CO), 21.9 (C-9), 25.7 (C-10), 32.0 (C-8), 38.1 (*Me*SO), 53.7 (C-11), 62.0 (C-6), 68.1 (C-4), 70.1 (C-2), 73.7 (C-3), 75.8 (C-5), 79.9 (C-1), 149.7 (C=N), 169.4, 169.5, 170.3, 170.7 (C=O). HR-ESI-MS *m*/*z* calcd for C_20_H_31_NO_11_S_2_ 525.1339, found 525.1331.

*S-(2,3,4,6-Tetra-O-acetyl-β-d-glucopyranosyl) (Z)-6-(methylsulfinyl)hexanethiohydroximate* *(***6d***).*

White amorphous powder (0.28 g, 51% yield), [α]_D_ -15 (*c* = 1.0, CHCl_3_). ^1^H NMR (CDCl_3_): δ 1.48–1.54 (m, 2H, H-10), 1.66–1.81 (m, 4H, H-9, H-11), 1.98, 2.01, 2.02, 2.05 (4s, 12H, *Me*CO), 2.47–2.55 (m, 2H, H-8), 2.58 (s, 3H, *Me*SO), 2.64–2.80 (m, 2H, H-12), 3.74 (ddd, 1H, *J*_4,5_ = 9.2, H-5), 4.09 (dd, 1H, *J*_5,6b_ = 2.6, *J*_6a,6b_ = 12.5, H-6b), 4.18 (dd, 1H, *J*_5,6a_ = 5.3, H-6a), 5.00–5.10 (m, 3H, H-4, H-1, H-2), 5.25 (ft, 1H, *J*_3,4_ = 9.1, H-3), 9.74 (bs, 1H, NOH). ^13^C NMR (CDCl_3_): δ 20.3, 20.5, 20.7 (*Me*CO), 22.1 (C-9), 26.3, 27.9 (C-10, C-11), 32.0 (C-8), 38.1 (*Me*SO), 53.9 (C-12), 62.0 (C-6), 68.3 (C-4), 69.9 (C-2), 73.6 (C-3), 75.7 (C-5), 79.8 (C-1), 150.3 (C=N), 169.3, 169.5, 170.3, 170.7 (C=O). HR-ESI-MS *m*/*z* calcd for C_21_H_33_NO_11_S_2_ 539.1495, found 539.1492.

*S-(2,3,4,6-Tetra-O-acetyl-β-d-glucopyranosyl) (Z)-8-(methylsulfinyl)octanethiohydroximate* *(***6e***).*

White amorphous powder (0.34 g, 60% yield), [α]_D_ -13 (*c* = 1.0, CHCl_3_). ^1^H NMR (CDCl_3_): δ 1.21–1.45 (m, 6H, H-10-H-12), 1.50–1.62 (m, 2H, H-9), 1.63–1.74 (m, 2H, H-13), 1.91, 1.93, 1.94, 1.97 (4s, 12H, *Me*CO), 2.39–2.47 (m, 2H, H-8), 2.55 (s, 3H, *Me*SO), 2.51–2.79 (m, 2H, H-14), 3.67 (ddd, 1H, *J*_4,5_ = 9.8, H-5), 3.99 (dd, 1H, *J*_5,6b_ = 2.5, *J*_6a,6b_ = 11.8, H-6b), 4.10 (dd, 1H, *J*_5,6a_ = 5.7, H-6a), 4.89–5.02 (m, 3H, H-4, H-1, H-2), 5.18 (ft, 1H, *J*_3,4_ = 9.9, H-3), 10.18 (bs, 1H, NOH). ^13^C NMR (CDCl_3_): δ 20.2, 20.3 (*Me*CO), 21.9, 26.4, 28.0, 28.1, 28.2 (C-9-C-13), 31.9 (C-8), 37.8 (*Me*SO), 53.9 (C-14), 62.0 (C-6), 67.9 (C-4), 69.9 (C-2), 73.5 (C-3), 75.5 (C-5), 79.6 (C-1), 150.1 (C=N), 169.1, 169.4, 170.1, 170.5 (C=O). HR-ESI-MS m/z calcd for C_23_H_37_NO_11_S_2_ 567.1808, found 567.1800.

*S-(2,3,4,6-Tetra-O-acetyl-β-d-glucopyranosyl) (Z)-10-(methylsulfinyl)decanethiohydroximate* *(***6f***).*

White amorphous powder (0.41 g, 68% yield), [α]_D_ -14 (*c* = 0.7, CHCl_3_).^1^H NMR (CDCl_3_): δ 1.25–1.41 (m, 10H, H-10-H-14), 1.49–1.65 (m, 4H, H-9, H-15), 1.99, 2.01, 2.02, 2.05 (4s, 12H, *Me*CO), 2.41–2.49 (m, 2H, H-8), 2.52 (s, 3H, *Me*SO), 2.51–2.77 (m, 2H, H-16), 3.72 (ddd, 1H, *J*_4,5_ = 10.0, H-5), 4.05 (dd, 1H, *J*_5,6b_ = 2.2, *J*_6a,6b_ = 12.5, H-6b), 4.26 (dd, 1H, *J*_5,6a_ = 5.5, H-6a), 5.04–5.13 (m, 3H, H-4, H-1, H-2), 5.24 (ft, 1H, *J*_3,4_ = 9.2, H-3), 8.90 (bs, 1H, NOH). ^13^C NMR (CDCl_3_): δ 20.3, 20.5, 20.6 (*Me*CO), 22.7, 26.0, 27.9, 28.0, 29.0, 29.1, 29.2 (C-9-C-15), 32.2 (C-8), 38.4 (*Me*SO), 53.6 (C-16), 62.0 (C-6), 68.6 (C-4), 70.5 (C-2), 73.7 (C-3), 75.4 (C-5), 79.0 (C-1), 150.7 (C=N), 169.4, 170.4, 170.7 (C=O). HR-ESI-MS *m*/*z* calcd for C_25_H_41_NO_11_S_2_ 595.2121, found 595.2111.

*S-(2,3,4,6-Tetra-O-acetyl-β-d-glucopyranosyl) (Z)-12-(methylsulfinyl)dodecanethiohydroximate* *(***6g***).*

White amorphous powder (0.41 g, 65% yield), [α]_D_ -15 (*c* = 0.8, CHCl_3_). ^1^H NMR (CDCl_3_): δ 1.33–1.47 (m, 14H, H-10-H-16), 1.50–1.68 (m, 4H, H-9, H-17), 1.98, 2.01, 2.02, 2.05 (4s, 12H, *Me*CO), 2.44–2.51 (m, 2H, H-8), 2.52 (s, 3H, *Me*SO), 2.53–2.81 (m, 2H, H-18), 3.74 (ddd, 1H, *J*_4,5_ = 10.0, H-5), 4.09 (dd, 1H, *J*_5,6b_ = 2.4, *J*_6a,6b_ = 12.3, H-6b), 4.17 (dd, 1H, *J*_5,6a_ = 5.5, H-6a), 5.01–5.15 (m, 3H, H-4, H-1, H-2), 5.21 (ft, 1H, *J*_3,4_ = 9.9, H-3), 10.5 (bs, 1H, NOH). ^13^C NMR (CDCl_3_): δ 20.2, 20.3, 20.4 (*Me*CO), 22.6, 26.8, 28.4, 29.6, 30.0 (C-9-C-17), 32.5 (C-8), 39.2 (*Me*SO), 53.0 (C-18), 62.1 (C-6), 67.7 (C-4), 70.2 (C-2), 73.4 (C-3), 75.7 (C-5), 79.8 (C-1), 151.3 (C=N), 169.4, 169.5, 170.3, 170.8 (C=O). HR-ESI-MS *m*/*z* calcd for C_27_H_45_NO_11_S_2_ 623.2434, found 623.2429.

##### Selective Oxidation of ω-Sulfides **5** to ω-Sulfones **7**

To a stirred solution of the protected glucosyl, thiohydroximate **5** (1 mmol) in methanol (40 mL) cooled at 0 °C, a solution of oxone (3 mmol) in water (10 mL) was added dropwise. After stirring at r.t. for 1–3 h, the white suspension was filtered, and the clear solution was diluted with water (50 mL) and then repeatedly extracted with chloroform (4 × 30 mL). The combined organic phases were dried over Na_2_SO_4_, filtered, and concentrated in vacuo. The residue was purified by flash column chromatography (dichloromethane containing 2–4% *v*/*v* methanol) to produce sulfones **7a**–**g** as amorphous solids.

*S-(2,3,4,6-Tetra-O-acetyl-β-d-glucopyranosyl) (Z)-3-(methylsulfonyl)propanethiohydroximate* *(***7a***).*

White amorphous powder (0.25 g, 49% yield), [α]_D_ -18 (*c* = 0.6, CHCl_3_). ^1^H NMR (CDCl_3_): δ 1.95, 1.98, 1.99, 2.04 (4s, 12H, *Me*CO), 2.93 (s, 3H, *Me*SO_2_), 2.94–3.12 (m, 2H, H-8), 3.27–3.48 (m, 2H, H-9), 3.81 (ddd, 1H, *J*_4,5_ = 10.0, H-5), 4.06 (dd, 1H, *J*_5,6b_ = 2.6, *J*_6a,6b_ = 12.5, H-6b), 4.25 (dd, 1H, *J*_5,6a_ = 4.6, H-6a), 4.95–5.10 (m, 3H, H-4, H-1, H-2), 5.21 (ft, 1H, *J*_3,4_ = 9.0, H-3), 9.5 (bs, 1H, NOH). ^13^C NMR (CDCl_3_): δ 20.2, 20.3, 20.4 (*Me*CO), 24.8 (C-8), 40.9 (*Me*SO_2_), 51.5 (C-9), 61.5 (C-6), 67.7 (C-4), 69.9 (C-2), 73.6 (C-3), 75.6 (C-5), 79.4 (C-1), 146.9 (C=N), 169.6, 169.7, 170.4, 171.1 (C=O). HR-ESI-MS *m*/*z* calcd for C_18_H_27_NO_12_S_2_ 513.0975, found 513.0969.

*S-(2,3,4,6-Tetra-O-acetyl-β-d-glucopyranosyl) (Z)-4-(methylsulfonyl)butanethiohydroximate* *(***7b***).*

White amorphous powder (0.29 g, 54% yield), [α]_D_ -15 (*c* = 0.7, CHCl_3_). ^1^H NMR (CDCl_3_): δ 1.95–2.23 (m, 2H, H-9), 1.99, 2.01, 2.03, 2.06 (4s, 12H, *Me*CO), 2.76 (t, 2H, *J* = 7.0, H-8), 2.93 (s, 3H, *Me*SO_2_), 3.01–3.17 (m, 2H, H-10), 3.84 (ddd, 1H, *J*_4,5_ = 10.5, H-5), 4.15 (dd, 1H, *J*_5,6b_ = 2.3, *J*_6a,6b_ = 11.8, H-6b), 4.21 (dd, 1H, *J*_5,6a_ = 5.2, H-6a), 4.99–5.15 (m, 3H, H-4, H-1, H-2), 5.26 (ft, 1H, *J*_3,4_ = 9.2, H-3), 9.2 (bs, 1H, NOH). ^13^C NMR (CDCl_3_): δ 20.4, 20.5, 20.6 (*Me*CO), 19.7 (C-9), 30.7 (C-8), 40.6 (*Me*SO_2_), 53.1 (C-10), 61.8 (C-6), 67.3 (C-4), 70.1 (C-2), 73.6 (C-3), 75.6 (C-5), 79.6 (C-1), 150.7 (C=N), 169.5, 169.6, 170.3, 170.9 (C=O). HR-ESI-MS *m*/*z* calcd for C_19_H_29_NO_12_S_2_ 527.1131, found 527.1124.

*S-(2,3,4,6-Tetra-O-acetyl-β-d-glucopyranosyl) (Z)-5-(methylsulfonyl)pentanethiohydroximate* *(***7c***).*

White amorphous powder (0.30 g, 56% yield), [α]_D_ -18 (*c* = 0.8, CHCl_3_). ^1^H NMR (CDCl_3_): δ 1.76–1.94 (m, 4H, H-9, H-10), 1.98, 2.00, 2.02, 2.05 (4s, 12H, *Me*CO), 2.53–2.60 (m, 2H, H-8), 2.91 (s, 3H, *Me*SO_2_), 3.06 (t, 2H, *J* = 7.3, H-11), 3.77 (ddd, 1H, *J*_4,5_ = 10.0, H-5), 4.11 (dd, 1H, *J*_5,6b_ = 2.1, *J*_6a,6b_ = 11.6, H-6b), 4.17 (dd, 1H, *J*_5,6a_ = 4.9, H-6a), 5.01–5.10 (m, 3H, H-4, H-1, H-2), 5.26 (ft, 1H, *J*_3,4_ = 9.4, H-3), 9.2 (bs, 1H, NOH). ^13^C NMR (CDCl_3_): δ 20.4, 20.5, 20.6 (*Me*CO), 21.3 (C-9), 25.2 (C-10), 31.6 (C-8), 40.6 (*Me*SO_2_), 53.9 (C-11), 62.0 (C-6), 68.0 (C-4), 70.1 (C-2), 73.6 (C-3), 75.7 (C-5), 79.7 (C-1), 151.0 (C=N), 169.4, 169.6, 170.3, 170.8 (C=O). HR-ESI-MS *m*/*z* calcd for C_20_H_31_NO_12_S_2_ 541.1288, found 541.1279.

*S-(2,3,4,6-Tetra-O-acetyl-β-d-glucopyranosyl) (Z)-6-(methylsulfonyl)hexanethiohydroximate* *(***7d***).*

White amorphous powder (0.33 g, 60% yield), [α]_D_ -13 (*c* = 0.8, CHCl_3_). ^1^H NMR (CDCl_3_): δ 1.47–1.55 (m, 2H, H-10), 1.61–1.69 (m, 2H, H-9), 1.78–1.89 (m, 2H, H-11), 1.96, 1.99, 2.00, 2.03 (4s, 12H, *Me*CO), 2.44–2.55 (m, 2H, H-8), 2.89 (s, 3H, *Me*SO_2_), 3.00 (t, 2H, *J* = 7.2, H-12), 3.74 (ddd, 1H, *J*_4,5_ = 9.5, H-5), 4.08 (dd, 1H, *J*_5,6b_ = 2.5, *J*_6a,6b_ = 12.3, H-6b), 4.18 (dd, 1H, *J*_5,6a_ = 5.1, H-6a), 4.95–5.09 (m, 3H, H-4, H-1, H-2), 5.24 (ft, 1H, *J*_3,4_ = 9.0, H-3), 9.2 (bs, 1H, NOH). ^13^C NMR (CDCl_3_): δ 20.4, 20.5, 20.6 (*Me*CO), 21.7 (C-9), 26.0, 27.3 (C-10, C-11), 31.8 (C-8), 40.6 (*Me*SO_2_), 54.0 (C-12), 62.0 (C-6), 68.0 (C-4), 70.1 (C-2), 73.5 (C-3), 75.6 (C-5), 79.7 (C-1), 151.6 (C=N), 169.4, 169.5, 170.3, 170.7 (C=O). HR-ESI-MS *m*/*z* calcd for C_21_H_33_NO_12_S_2_ 555.1444, found 555.1440.

*S-(2,3,4,6-Tetra-O-acetyl-β-d-glucopyranosyl) (Z)-8-(methylsulfonyl)octanethiohydroximate* *(***7e***).*

White amorphous powder (0.40 g, 68% yield), [α]_D_ -14 (*c* = 0.8, CHCl_3_). ^1^H NMR (CDCl_3_): δ 1.24–1.49 (m, 6H, H-10-H-12), 1.55–1.68 (m, 2H, H-9), 1.73–1.89 (m, 2H, H-13), 1.96, 1.99, 2.02, 2.05 (4s, 12H, *Me*CO), 2.42–2.58 (m, 2H, H-8), 2.88 (s, 3H, *Me*SO_2_), 2.99 (t, 2H, *J* = 7.0, H-14), 3.64 (ddd, 1H, *J*_4,5_ = 10.2, H-5), 4.08 (dd, 1H, *J*_5,6b_ = 2.4, *J*_6a,6b_ = 12.3, H-6b), 4.17 (dd, 1H, *J*_5,6a_ = 5.3, H-6a), 5.00–5.09 (m, 3H, H-4, H-1, H-2), 5.26 (ft, 1H, *J*_3,4_ = 9.4, H-3), 9.0 (bs, 1H, NOH). ^13^C NMR (CDCl_3_): δ 20.3, 20.5, 20.6 (*Me*CO), 21.8 (C-9), 26.4, 27.8, 28.2, 28.3 (C-10-C-13), 32.0 (C-8), 40.3 (*Me*SO_2_), 54.4 (C-14), 62.1 (C-6), 68.0 (C-4), 70.0 (C-2), 73.6 (C-3), 75.7 (C-5), 79.8 (C-1), 151.9 (C=N), 169.3, 169.5, 170.3, 170.7 (C=O). HR-ESI-MS *m*/*z* calcd for C_23_H_37_NO_12_S_2_ 583.1756, found 583.1749.

*S-(2,3,4,6-Tetra-O-acetyl-β-d-glucopyranosyl) (Z)-10-(methylsulfonyl)decanethiohydroximate* *(***7f***).*

White amorphous powder (0.40 g, 65% yield), [α]_D_ -14 (*c* = 0.7, CHCl_3_). ^1^H NMR (CDCl_3_): δ 1.22–1.41 (m, 10H, H-10-H-14), 1.41–1.57 (m, 2H, H-9), 1.69–1.85 (m, 2H, H-15), 1.98, 2.01, 2.03, 2.06 (4s, 12H, *Me*CO), 2.42–2.47 (m, 2H, H-8), 2.87 (s, 3H, *Me*SO_2_), 2.96 (t, 2H, *J* = 7.2, H-16), 3.72 (ddd, 1H, *J*_4,5_ = 10.1, H-5), 4.16 (dd, 1H, *J*_5,6b_ = 2.6, *J*_6a,6b_ = 12.1, H-6b), 4.26 (dd, 1H, *J*_5,6a_ = 5.6, H-6a), 5.08–5.19 (m, 3H, H-4, H-1, H-2), 5.22 (ft, 1H, *J*_3,4_ = 9.8, H-3), 9.80 (bs, 1H, NOH). ^13^C NMR (CDCl_3_): δ 20.3, 20.5, (*Me*CO), 21.7 (C-9), 26.2, 27.9, 29.4, 29.5, 29.9 (C-10-C-15), 32.8 (C-8), 40.7 (*Me*SO_2_), 54.0 (C-16), 61.9 (C-6), 68.2 (C-4), 68.5 (C-2), 73.6 (C-3), 76.1 (C-5), 80.0 (C-1), 151.4 (C=N), 169.5, 169.8, 170.8, 171.0 (C=O). HR-ESI-MS *m*/*z* calcd for C_25_H_41_NO_12_S_2_ 611.2068, found 611.2061.

*S-(2,3,4,6-Tetra-O-acetyl-β-d-glucopyranosyl) (Z)-12-(methylsulfonyl)dodecanethiohydroximate* *(***7g***).*

White amorphous powder (0.45 g, 70% yield), [α]_D_ -13 (*c* = 0.8, CHCl_3_). ^1^H NMR (CDCl_3_): δ 1.33–1.51 (m, 14H, H-10-H-16), 1.53–1.87 (m, 4H, H-9, H-17), 1.99, 2.01, 2.02, 2.05 (4s, 12H, *Me*CO), 2.40–2.59 (m, 2H, H-8), 2.89 (s, 3H, *Me*SO_2_), 2.99 (t, 2H, *J* = 7.1, H-18), 3.77 (ddd, 1H, *J*_4,5_ = 10.0, H-5), 4.12 (dd, 1H, *J*_5,6b_ = 2.5, *J*_6a,6b_ = 12.5, H-6b), 4.23 (dd, 1H, *J*_5,6a_ = 5.4, H-6a), 4.98–5.20 (m, 3H, H-4, H-1, H-2), 5.26 (ft, 1H, *J*_3,4_ = 9.4, H-3), 8.66 (bs, 1H, NOH). ^13^C NMR (CDCl_3_): δ 20.1, 20.4, 20.5 (*Me*CO), 22.6, 27.1, 28.6, 28.8, 29.6, 29.8, 30.3 (C-9-C-17), 32.6 (C-8), 40.7 (*Me*SO_2_), 54.6 (C-18), 62.1 (C-6), 67.3 (C-4), 70.7 (C-2), 73.9 (C-3), 75.9 (C-5), 79.6 (C-1), 152.2 (C=N), 169.7, 170.0, 170.4, 170.7 (C=O). HR-ESI-MS *m*/*z* calcd for C_27_H_45_NO_12_S_2_ 639.2383, found 639.2375.

#### 3.2.2. Pathway 1 and 2 Common Procedures

##### General Procedure for NO-Sulfation of the Glucosyl Thiohydroximates **6** and **7**

A solution of compound **6** or **7** was treated with sulfur trioxide pyridine complex as previously described in detail [29]. The obtained *NO*-sulfated compounds **8** or **9** after purification are described as follows.

*Per-O-acetylated 2-methylsulfinylethyl glucosinolate* *(***8a***).*

White amorphous powder (0.14 g, 56% yield), [α]_D_ -21 (*c* = 1.0, MeOH). ^1^H NMR (DMSO-*d6*): δ 1.96, 1.98, 2.01, 2.02 (4s, 12H, *Me*CO), 2.58 (s, 3H, *Me*SO), 2.59–2.82 (m, 4H, H-8, H-9), 4.10–4.21 (m, 3H, H-5, H-6b, H-6a), 4.87 (t, 1H, *J*_2,3_ = 9.6, H-2), 4.92 (t, 1H, *J*_4,5_ = 8.8, H-4), 5.43 (t, 1H, *J*_3,4_ = 9.5, H-3), 5.58 (d, 1H, *J*_1,2_ = 10.0, H-1). ^13^C NMR (DMSO-*d6*): δ 20.1, 20.2, 20.3, 20.5 (*Me*CO), 25.6 (C-8), 38.0 (*Me*SO), 54.2 (C-9), 61.9 (C-6), 69.4 (C-4), 71.9 (C-2), 74.6 (C-3), 77.9 (C-5), 79.5 (C-1), 153.2 (C=N), 169.5, 170.2, 170.3 (C=O).

*Per-O-acetylated 3-methylsulfinylpropyl glucosinolate* *(***8b***).*

White amorphous powder (0.14 g, 54% yield), [α]_D_ -19 (*c* = 0.9, MeOH). ^1^H NMR (DMSO-*d6*): δ 1.83–2.02 (m, 14H, H-9, *Me*CO), 2.58 (s, 3H, *Me*SO), 2.68–2.90 (m, 4H, H-8, H-10), 4.00–4.16 (m, 3H, H-5, H-6a, H-6b), 4.86 (t, 1H, *J*_2,3_ = 9.6, H-2), 4.93 (t, 1H, *J*_4,5_ = 8.0, H-4), 5.37 (t, 1H, *J*_3,4_ = 9.3, H-3), 5.51 (d, 1H, *J*_1,2_ = 10.2, H-1). ^13^C NMR (DMSO-*d6*): δ 20.0, 20.2, 20.3, 20.5 (*Me*CO), 20.7 (C-9), 32.1 (C-8), 38.8 (*Me*SO), 54.2 (C-10), 61.9 (C-6), 69.5 (C-4), 72.8 (C-2), 74.4 (C-3), 78.2 (C-5), 79.3 (C-1), 153.3 (C=N), 169.4, 169.5, 169.8, 170.3 (C=O).

*Per-O-acetylated 4-methylsulfinylbutyl glucosinolate* *(***8c***).*

White amorphous powder (0.15 g, 58% yield), [α]_D_ -15 (*c* = 0.6, MeOH). ^1^H NMR (DMSO-*d6*): δ 1.65–1.82 (m, 4H, H-9, H-10), 1.97, 2.02, 2.03, 2.05 (4s, 12H, *Me*CO), 2.59 (s, 3H, *Me*SO), 2.58–2.89 (m, 4H, H-8, H-11), 4.02–4.21 (m, 3H, H-5, H-6a, H-6b), 4.88 (t, 1H, *J*_2,3_ = 9.6, H-2), 4.93 (t, 1H, *J*_4,5_ = 8.3, H-4), 5.45 (t, 1H, *J*_3,4_ = 9.5, H-3), 5.52 (d, 1H, *J*_1,2_ = 10.0, H-1). ^13^C NMR (DMSO-*d6*): δ 20.5, 20.6, 20.8 (*Me*CO), 22.4 (C-9), 26.5 (C-10), 32.7 (C-8), 37.9 (*Me*SO), 53.9 (C-11), 63.2 (C-6), 69.4 (C-4), 71.3 (C-2), 74.9 (C-3), 76.5 (C-5), 80.6 (C-1), 159.5 (C=N), 171.3, 171.5, 171.7, 172.6 (C=O).

*Per-O-acetylated 5-methylsulfinylpentyl glucosinolate* *(***8d***).*

White amorphous powder (0.16 g, 62% yield), [α]_D_ -16 (*c* = 0.7, MeOH). ^1^H NMR (CD_3_OD): δ 1.54–1.68 (m, 2H, H-10), 1.73–1.88 (m, 4H, H-9, H-11), 1.97, 2.02, 2.03, 2.06 (4s, 12H, *Me*CO), 2.67 (s, 3H, *Me*SO), 2.72 (t, 2H, *J*_8,9_ = 7.2, H-8), 2.79–2.95 (m, 2H, H-12), 4.05 (ddd, 1H, *J*_4,5_ = 9.6, H-5), 4.15 (dd, 1H, *J*_5,6b_ = 2.7, *J*_6a,6b_ = 12.4, H-6b), 4.23 (dd, 1H, *J*_5,6a_ = 5.1, H-6a), 4.98 (t, 1H, *J*_2,3_ = 9.2, H-2), 5.05 (t, 1H, *J*_4,5_ = 8.9, H-4), 5.39–5.43 (m, 2H, H-1, H-3). ^13^C NMR (CD_3_OD): δ 20.5, 20.7 (*Me*CO), 23.0 (C-10), 27.3 (C-9), 28.6 (C-11), 33.0 (C-8), 38.0 (*Me*SO), 54.5 (C-12), 63.3 (C-6), 69.5 (C-4), 71.4 (C-2), 75.0 (C-3), 76.7 (C-5), 80.8 (C-1), 159.7 (C=N), 171.1, 171.5, 171.7, 172.4 (C=O).

*Per-O-acetylated 7-methylsulfinylheptyl glucosinolate* *(***8e***).*

White amorphous powder (0.21 g, 75% yield), [α]_D_ -16 (*c* = 0.9, MeOH). ^1^H NMR (DMSO-*d6*): δ 1.22–1.41 (m, 6H, H-10-H-12), 1.42–1.60 (m, 4H, H-9, H-13), 1.96, 1.98, 1.99, 2.03 (4s, 12H, *Me*CO), 2.55 (s, 3H, *Me*SO), 2.56–2.88 (m, 4H, H-8, H-14), 4.05–4.21 (m, 3H, H-5, H-6b, H-6a), 4.82 (t, 1H, *J*_2,3_ = 9.4, H-2), 4.93 (t, 1H, *J*_4,5_ = 8.9, H-4), 5.47 (t, 1H, *J*_3,4_ = 9.6, H-3), 5.50 (d, 1H, *J*_1,2_ = 10.1, H-1). ^13^C NMR (DMSO-*d6*): δ 20.3, 20.4, 20.5 (*Me*CO), 22.1, 26.5, 28.1, 28.2, 28.3 (C-9-C-13), 31.7 (C-8), 37.6 (*Me*SO), 54.0 (C-14), 62.3 (C-6), 68.5 (C-4), 70.1 (C-2), 73.2 (C-3), 74.7 (C-5), 78.7 (C-1), 158.5 (C=N), 169.4, 169.5, 169.9, 170.2 (C=O).

*Per-O-acetylated 9-methylsulfinylnonyl glucosinolate* *(***8f***).*

White amorphous powder (0.22 g, 78% yield), [α]_D_ -16 (*c* = 1.0, MeOH). ^1^H NMR (DMSO-*d6*): δ 1.19–1.40 (m, 10H, H-10-H-14), 1.42–1.57 (m, 4H, H-9, H-15), 1.98, 1.99, 2.01, 2.04 (4s, 12H, *Me*CO), 2.59 (s, 3H, *Me*SO), 2.58–2.92 (m, 4H, H-8, H-16), 3.99–4.18 (m, 3H, H-5, H-6b, H-6a), 4.89 (t, 1H, *J*_2,3_ = 9.5, H-2), 4.91 (t, 1H, *J*_4,5_ = 8.7, H-4), 5.43 (t, 1H, *J*_3,4_ = 9.7, H-3), 5.51 (d, 1H, *J*_1,2_ = 10.1, H-1). ^13^C NMR (DMSO-*d6*): δ 20.2, 20.3, 20.4 (*Me*CO), 21.9, 26.4, 28.2, 28.8, 29.1, 29.4 (C-9-C-15), 32.4 (C-8), 38.2 (*Me*SO), 53.8 (C-16), 62.2 (C-6), 68.6 (C-4), 70.4 (C-2), 72.9 (C-3), 74.7 (C-5), 78.3 (C-1), 159.7 (C=N), 171.3, 171.5, 171.8, 172.4 (C=O).

*Per-O-acetylated 11-methylsulfinylundecyl glucosinolate* *(***8g***).*

White amorphous powder (0.23 g, 76% yield), [α]_D_ -18 (*c* = 1.1, MeOH). ^1^H NMR (DMSO-*d6*): δ 1.22–1.45 (m, 14H, H-10-H-16), 1.47–1.68 (m, 4H, H-9, H-17), 1.97, 1.98, 2.00, 2.03 (4s, 12H, *Me*CO), 2.58 (s, 3H, *Me*SO), 2.59–2.86 (m, 4H, H-8, H-18), 4.00–4.26 (m, 3H, H-5, H-6b, H-6a), 4.90 (t, 1H, *J*_2,3_ = 9.4, H-2), 4.93 (t, 1H, *J*_4,5_ = 8.8, H-4), 5.43 (t, 1H, *J*_3,4_ = 9.5, H-3), 5.51 (d, 1H, *J*_1,2_ = 10.0, H-1). ^13^C NMR (DMSO-*d6*): δ 20.2, 20.4, 20.5 (*Me*CO), 22.8, 26.6, 28.4, 28.9, 30.1, 30.3, 30.4 (C-9-C-17), 32.9 (C-8), 39.6 (*Me*SO), 53.6 (C-18), 62.6 (C-6), 68.4 (C-4), 70.1 (C-2), 73.3 (C-3), 75.1 (C-5), 78.6 (C-1), 159.4 (C=N), 171.2, 171.4, 171.7, 172.2 (C=O).

*Per-O-acetylated 2-methylsulfonylethyl glucosinolate* *(***9a***).*

White amorphous powder (0.15 g, 58% yield), [α]_D_ -19 (*c* = 0.9, MeOH). ^1^H NMR (DMSO-*d6*): δ 1.97, 1.98, 2.02, 2.05 (4s, 12H, *Me*CO), 2.56 (t, 2H, *J*_8.9_ = 7.3, H-8), 2.96 (s, 3H, *Me*SO_2_), 3.17 (t, 2H, *J*_9,8_ = 7.6, H-9), 4.12–4.23 (m, 3H, H-5, H-6b, H-6a), 4.85 (t, 1H, *J*_2,3_ = 9.4, H-2), 4.91 (t, 1H, *J*_4,5_ = 9.8, H-4), 5.44 (t, 1H, *J*_3,4_ = 9.4, H-3), 5.54 (d, 1H, *J*_1,2_ = 10.0, H-1). ^13^C NMR (DMSO-*d6*): δ 20.0, 20.4, 20.5 (*Me*CO), 25.8 (C-8), 40.5 (*Me*SO_2_), 54.1 (C-9), 61.4 (C-6), 67.6 (C-4), 70.0 (C-2), 73.4 (C-3), 75.6 (C-5), 79.4 (C-1), 154.9 (C=N), 169.5, 170.1, 170.3 (C=O).

*Per-O-acetylated 3-methylsulfonylpropyl glucosinolate* *(***9b***).*

White amorphous powder (0.14 g, 56% yield), [α]_D_ -21 (*c* = 1.0, MeOH). ^1^H NMR (DMSO-*d6*): δ 1.92–2.03 (m, 2H, H-9), 1.94, 1.97, 1.99, 2.01 (4s, 12H, *Me*CO), 2.71 (t, 2H, *J*_8.9_ = 7.1, H-8), 2.97 (s, 3H, *Me*SO_2_), 3.14–3.22 (m, 2H, H-10), 4.04–4.15 (m, 3H, H-5, H-6a, H-6b), 4.87 (t, 1H, *J*_2,3_ = 9.1, H-2), 4.94 (t, 1H, *J*_4,5_ = 8.5, H-4), 5.39 (t, 1H, *J*_3,4_ = 9.9, H-3), 5.50 (d, 1H, *J*_1,2_ = 10.6, H-1). ^13^C NMR (DMSO-*d6*): δ 20.1, 20.4, 20.6 (*Me*CO), 22.4 (C-9), 30.5 (C-8), 41.0 (*Me*SO_2_), 53.8 (C-10), 61.5 (C-6), 69.1 (C-4), 71.9 (C-2), 74.6 (C-3), 77.7 (C-5), 79.9 (C-1), 158.0 (C=N), 169.4, 169.5, 170.2, 170.4 (C=O).

*Per-O-acetylated 4-methylsulfonylbutyl glucosinolate* *(***9c***).*

White amorphous powder (0.16 g, 61% yield), [α]_D_ -16 (*c* = 0.9, MeOH). ^1^H NMR (DMSO-*d6*): δ 1.51–1.84 (m, 4H, H-9, H-10), 1.97, 1.99, 2.00, 2.03 (4s, 12H, *Me*CO), 2.59 (t, 2H, *J*_8.9_ = 7.4, H-8), 2.92 (s, 3H, *Me*SO_2_), 3.16 (t, 2H, *J* = 7.7, H-11), 3.95–4.22 (m, 3H, H-5, H-6a, H-6b), 4.90 (t, 1H, *J*_2,3_ = 9.4, H-2), 4.95 (t, 1H, *J*_4,5_ = 8.6, H-4), 5.48 (t, 1H, *J*_3,4_ = 9.8, H-3), 5.51 (d, 1H, *J*_1,2_ = 10.2, H-1). ^13^C NMR (DMSO-*d6*): δ 20.4, 20.5, 20.8 (*Me*CO), 22.1 (C-9), 25.8 (C-10), 31.7 (C-8), 40.8 (*Me*SO_2_), 54.8 (C-11), 63.5 (C-6), 69.3 (C-4), 71.3 (C-2), 74.7 (C-3), 76.8 (C-5), 80.9 (C-1), 158.7 (C=N), 170.7, 171.0, 172.4, 172.5 (C=O).

*Per-O-acetylated 5-methylsulfonylpentyl glucosinolate* *(***9d***).*

White amorphous powder (0.17 g, 64% yield), [α]_D_ -15 (*c* = 0.8, MeOH). ^1^H NMR (CD_3_OD): δ 1.53–1.65 (m, 2H, H-10), 1.73–1.91 (m, 4H, H-9, H-11), 1.98, 2.02, 2.03, 2.06 (4s, 12H, *Me*CO), 2.70 (t, 2H, *J*_8.9_ = 7.0, H-8), 2.97 (s, 3H, *Me*SO_2_), 3.16 (t, 2H, *J* = 7.8, H-12), 4.02–4.09 (m, 1H, H-5), 4.15 (dd, 1H, *J*_5,6b_ = 2.2, *J*_6a,6b_ = 12.1, H6b), 4.22 (dd, 1H, *J*_5,6a_ = 5.1, H6a), 4.98 (t, 1H, *J*_2,3_ = 9.5, H-2), 5.05 (t, 1H, *J*_4,5_ = 9.8, H-4), 5.39–5.43 (m, 2H, H-1, H-3). ^13^C NMR (CD_3_OD): δ 20.5, 20.7 (*Me*CO), 22.8 (C-10), 27.2 (C-9), 28.3 (C-11), 32.9 (C-8), 40.7 (*Me*SO_2_), 54.9 (C-12), 63.3 (C-6), 69.4 (C-4), 71.3 (C-2), 74.9 (C-3), 76.6 (C-5), 80.7 (C-1), 159.8 (C=N), 171.1, 171.4, 171.7, 172.4 (C=O).

*Per-O-acetylated 7-methylsulfonylheptyl glucosinolate* *(***9e***).*

White amorphous powder (0.21 g, 76% yield), [α]_D_ -18 (*c* = 1.0, MeOH). ^1^H NMR (DMSO-*d6*): δ 1.25–1.41 (m, 6H, H-10-H-12), 1.42–1.65 (m, 4H, H-9, H-13), 1.97, 1.98, 1.99, 2.02 (4s, 12H, *Me*CO), 2.58 (t, 2H, *J*_8.9_ = 7.4, H-8), 2.95 (s, 3H, *Me*SO_2_), 3.16 (t, 2H, *J* = 7.9, H-14), 4.03–4.20 (m, 3H, H-5, H-6b, H-6a), 4.89 (t, 1H, *J*_2,3_ = 9.5, H-2), 4.91 (t, 1H, *J*_4,5_ = 8.7, H-4), 5.43 (t, 1H, *J*_3,4_ = 9.7, H-3), 5.51 (d, 1H, *J*_1,2_ = 10.1, H-1). ^13^C NMR (DMSO-*d6*): δ 20.3, 20.4 (*Me*CO), 21.5, 26.8, 28.0, 28.5, 28.6 (C-9-C-13), 32.2 (C-8), 40.6 (*Me*SO_2_), 54.3 (C-14), 61.5 (C-6), 69.3 (C-4), 71.3 (C-2), 75.2 (C-3), 76.5 (C-5), 80.4 (C-1), 159.8 (C=N), 171.0, 171.3, 171.7, 172.0 (C=O).

*Per-O-acetylated 9-methylsulfonylnonyl glucosinolate* *(***9f***).*

White amorphous powder (0.25 g, 85% yield), [α]_D_ -16 (*c* = 0.9, MeOH). ^1^H NMR (DMSO-*d6*): δ 1.21–1.40 (m, 10H, H-10-H-14), 1.42–1.57 (m, 4H, H-9, H-15), 1.98, 1.99, 2.02, 2.04 (*Me*CO), 2.49–2.61 (m, 2H, H-8), 2.96 (s, 3H, *Me*SO_2_), 3.18 (t, 2H, *J* = 7.6, H-16), 3.99–4.18 (m, 3H, H-5, H-6b, H-6a), 4.81 (t, 1H, *J*_2,3_ = 9.4, H-2), 4.93 (t, 1H, *J*_4,5_ = 8.9, H-4), 5.47 (t, 1H, *J*_3,4_ = 9.6, H-3), 5.50 (d, 1H, *J*_1,2_ = 10.1, H-1). ^13^C NMR (DMSO-*d6*): δ 20.2, 20.3, 20.4 (*Me*CO), 21.6, 26.4, 28.1, 29.6, 29.7, 30.0 (C-9-C-15), 33.2 (C-8), 40.9 (*Me*SO_2_), 54.3 (C-16), 62.2 (C-6), 69.5 (C-4), 71.4 (C-2), 74.7 (C-3), 76.5 (C-5), 79.9 (C-1), 159.6 (C=N), 171.2, 171.6, 171.9, 172.5 (C=O).

*Per-O-acetylated 11-methylsulfonylundecyl glucosinolate* *(***9g***).*

White amorphous powder (0.25 g, 82% yield), [α]_D_ -17 (*c* = 1.0, MeOH). ^1^H NMR (DMSO-*d6*): δ 1.22–1.42 (m, 14H, H-10-H-16), 1.47–1.68 (m, 4H, H-9, H-17), 1.97, 1.99, 2.00, 2.02 (*Me*CO), 2.53–2.64 (m, 2H, H-8), 2.98 (s, 3H, *Me*SO_2_), 3.16 (t, 2H, *J* = 7.8, H-18), 4.01–4.25 (m, 3H, H-5, H-6b, H-6a), 4.86 (t, 1H, *J*_2,3_ = 9.3, H-2), 4.91 (t, 1H, *J*_4,5_ = 9.2, H-4), 5.44 (t, 1H, *J*_3,4_ = 9.5, H-3), 5.52 (d, 1H, *J*_1,2_ = 10.2, H-1). ^13^C NMR (DMSO-*d6*): δ 20.2, 20.4, 20.5 (*Me*CO), 22.3, 27.5, 28.7, 28.9, 29.6, 29.8, 31.0 (C-9-C-17), 32.7 (C-8), 40.5 (*Me*SO_2_), 54.0 (C-18), 63.3 (C-6), 70.4 (C-4), 71.4 (C-2), 75.0 (C-3), 77.3 (C-5), 80.4 (C-1), 160.3 (C=N), 171.3, 171.5, 171.9, 172.6 (C=O).

##### General Procedure for Deprotection of the Glucopyranosyl Moiety

Peracetylated GSLs **8** or **9** were transformed into GSLs **3** or **4** using the general de-*O*-acetylation procedure previously described in full detail [29].

*2-(Methylsulfinyl)ethyl glucosinolate* *(***3a***)* [443340-10-1].

White amorphous powder (0.084 g, 82% yield), [α]_D_ -23 (*c* = 0.7, H_2_O). ^1^H NMR (D_2_O): δ 2.54 (s, 3H, *Me*SO), 2.85–2.91 (m, 2H, H-8), 2.99–3.06 (m, 2H, H-9), 3.38–3.46 (m, 2H, H-2, H-4), 3.53–3.62 (m, 2H, H-5, H-3), 3.71 (dd, 1H, *J*_5,6b_ = 5.7, *J*_6a,6b_ = 12.9, H-6b), 3.88 (dd, 1H, *J*_5,6a_ = 2.5, H-6a), 5.02 (d, 1H, *J*_1,2_ = 10.3, H-1). ^13^C NMR (D_2_O): δ 25.5 (C-8), 37.9 (*Me*SO), 52.7 (C-9), 62.4 (C-6), 70.6 (C-4), 73.7 (C-2), 79.0 (C-3), 81.8 (C-5), 83.9 (C-1), 165.1 (C=N). HR-ESI-MS *m*/*z* calcd for C_10_H_18_NO_10_S_3_ [M−H]^−^ 408.0093, found 408.0084.

*3-(Methylsulfinyl)propyl glucosinolate* *(***3b***). Glucoiberin* [554-88-1].

White amorphous powder (0.085 g, 80% yield), [α]_D_ -19 (*c* = 0.7, H_2_O). ^1^H NMR (D_2_O): δ 1.96–2.06 (m, 2H, H-9), 2.56 (s, 3H, *Me*SO), 2.77 (t, 2H, *J* = 6.8, H-8), 2.82 (t, 2H, *J* = 6.8, H-10), 3.25–3.32 (m, 2H, H-2, H-4), 3.39–3.46 (m, 2H, H-5, H-3), 3.55 (dd, 1H, *J*_5,6b_ = 5.7, *J*_6a,6b_ = 12.5, H-6b), 3.74 (dd, 1H, *J*_5,6a_ = 2.6, H-6a), 4.91 (d, 1H, *J*_1,2_ = 10.0, H-1). ^13^C NMR (D_2_O): δ 21.7 (C-9), 32.8 (C-8), 38.8 (*Me*SO), 54.2 (C-10), 61.8 (C-6), 70.4 (C-4), 73.2 (C-2), 78.6 (C-3), 81.6 (C-5), 83.7 (C-1), 165.2 (C=N) [33]. HR-ESI-MS *m*/*z* calcd for C_11_H_20_NO_10_S_3_ [M−H]^−^ 422.0249, found 422.0239.

*4-(Methylsulfinyl)butyl glucosinolate* *(***3c***). Glucoraphanin* [21414-41-5].

White amorphous powder (0.092 g, 84% yield), [α]_D_ -19 (*c* = 0.7, H_2_O). ^1^H NMR (D_2_O): δ 1.72–1.98 (m, 4H, H-9, H-10), 2.58 (s, 3H, *Me*SO), 2.56–2.88 (m, 4H, H-8, H-11), 3.40–3.49 (m, 2H, H-2, H-4), 3.55–3.64 (m, 2H, H-5, H-3), 3.69 (dd, 1H, *J*_5,6b_ = 5.8, *J*_6a,6b_ = 13.2, H-6b), 3.90 (dd, 1H, *J*_5,6a_ = 2.6, H-6a), 5.00 (d, 1H, *J*_1,2_ = 10.1, H-1). ^13^C NMR (D_2_O): δ 22.7, 26.9, 32.2, 37.9 (C-8-C-11), 54.0 (*Me*SO), 61.9, 71.3, 73.5, 79.4, 82.4, 83.3 (C-1-C-6), 164.8 (*C*=N). [34]. HR-ESI-MS *m*/*z* calcd for C_12_H_22_NO_10_S_3_ [M−H]^−^ 436.0406, found 436.0397.

*5-(Methylsulfinyl)pentyl glucosinolate* *(***3d***). Glucoalyssin* [499-37-6].

White amorphous powder (0.098 g, 87% yield), [α]_D_ -19 (*c* = 0.8, H_2_O). ^1^H NMR (D_2_O): δ 1.52–1.65 (m, 2H, H-10), 1.74–1.90 (m, 4H, H-9, H-11), 2.60 (s, 3H, *Me*SO), 2.68–2.75 (m, 2H, H-8), 2.75–2.93 (m, 2H, H-12), 3.42–3.48 (m, 2H, H-2, H-4), 3.53–3.59 (m, 2H, H-5, H-3), 3.72 (dd, 1H, *J*_5,6b_ = 5.8, *J*_6a,6b_ = 12.8, H-6b), 3.80 (dd, 1H, *J*_5,6a_ = 2.7, H-6a), 5.08 (d, 1H, *J*_1,2_ = 10.0, H-1). ^13^C NMR (D_2_O): δ 22.3, 27.4, 29.0, 32.7, 38.2 (C-8-C-12), 54.9 (*Me*SO), 61.9, 70.5, 73.3, 78.9, 81.3, 83.6 (C-1-C-6), 164.9 (*C*=N). [35]. HR-ESI-MS *m*/*z* calcd for C_13_H_24_NO_10_S_3_ [M−H]^−^ 450.0562, found 450.0553.

*7-(Methylsulfinyl)heptyl glucosinolate* *(***3e***). Glucoibarin* [112572-51-7].

White amorphous powder (0.108 g, 91% yield), [α]_D_ -21 (*c* = 0.9, H_2_O). ^1^H NMR (D_2_O): δ 1.20–1.44 (m, 6H, H-10-H-12), 1.47–1.61 (m, 4H, H-9, H-13), 2.54 (s, 3H, *Me*SO), 2.55–2.86 (m, 4H, H-8, H-14), 3.45–3.51 (m, 2H, H-2, H-4), 3.54–3.68 (m, 2H, H-5, H-3), 3.69 (dd, 1H, *J*_5,6b_ = 5.7, *J*_6a,6b_ = 12.5, H-6b), 3.85 (dd, 1H, *J*_5,6a_ = 2.6, H-6a), 4.97 (d, 1H, *J*_1,2_ = 10.0, H-1). ^13^C NMR (D_2_O): δ 21.8, 26.5, 28.0, 28.2, 32.1, 37.9 (C-8-C-14), 54.5 (*Me*SO), 61.2, 70.5, 73.1, 78.9, 81.7, 83.3 (C-1-C-6), 164.6 (*C*=N). HR-ESI-MS *m*/*z* calcd for C_15_H_28_NO_10_S_3_ [M−H]^−^ 478.0874, found 478.0862.

*9-(Methylsulfinyl)nonyl glucosinolate* *(***3f***). Glucoarabin* [67920-64-3].

White amorphous powder (0.117 g, 93% yield), [α]_D_ -21 (*c* = 1.0, H_2_O). ^1^H NMR (D_2_O): δ 1.22–1.43 (m, 10H, H-10-H-14), 1.45–1.59 (m, 4H, H-9, H-15), 2.57 (s, 3H, *Me*SO), 2.58–2.91 (m, 4H, H-8, H-16), 3.39–3.47 (m, 2H, H-2, H-4), 3.55–3.67 (m, 2H, H-5, H-3), 3.71 (dd, 1H, *J*_5,6b_ = 5.8, *J*_6a,6b_ = 12.8, H-6b), 3.90 (dd, 1H, *J*_5,6a_ = 2.6, H-6a), 5.07 (d, 1H, *J*_1,2_ = 10.1, H-1). ^13^C NMR (D_2_O): δ 22.9, 26.8, 28.1, 28.9, 29.2 (br), 29.5, 32.7, 38.0 (C-8-C-16), 54.4 (*Me*SO), 61.7, 70.7, 73.3, 78.3, 82.0, 83.4 (C-1-C-6), 164.9 (*C*=N) [36,37]. HR-ESI-MS *m*/*z* calcd for C_17_H_32_NO_10_S_3_ [M−H]^−^ 506.1187, found 506.1180.

*11-(Methylsulfinyl)undecyl glucosinolate* *(***3g***)* [186037-18-3].

White amorphous powder (0.121 g, 92% yield), [α]_D_ -19 (*c* = 0.7, H_2_O). ^1^H NMR (D_2_O): δ 1.21–1.40 (m, 14H, H-10-H-16), 1.42–1.68 (m, 4H, H-9, H-17), 2.58 (s, 3H, *Me*SO), 2.59–2.85 (m, 4H, H-8, H-18), 3.39–3.48 (m, 2H, H-2, H-4), 3.57–3.69 (m, 2H, H-5, H-3), 3.68 (dd, 1H, *J*_5,6b_ = 5.7, *J*_6a,6b_ = 13.0, H-6b), 3.90 (dd, 1H, *J*_5,6a_ = 2.6, H-6a), 5.06 (d, 1H, *J*_1,2_ = 10.0, H-1). ^13^C NMR (D_2_O): δ 22.6, 26.9, 28.7br, 28.9, 29.6 (br), 29.9, 30.0, 32.7, 39.7 (C-8-C-18), 54.3 (*Me*SO), 62.3, 70.1, 73.4, 78.9, 81.6, 83.0 (C-1-C-6), 165.1 (*C*=N). HR-ESI-MS *m*/*z* calcd for C_19_H_36_NO_10_S_3_ [M−H]^−^ 534.1501, found 534.1497.

*2-(Methylsulfonyl)ethyl glucosinolate* *(***4a***).*

White amorphous powder (0.085 g, 80% yield), [α]_D_ -21 (*c* = 0.8, H_2_O). ^1^H NMR (D_2_O): δ 2.58 (t, 2H, *J* = 7.6, H-8), 2.95 (s, 3H, *Me*SO_2_), 3.17 (t, 2H, *J* = 7.5, H-9), 3.42–3.51 (m, 2H, H-2, H-4), 3.54–3.67 (m, 2H, H-5, H-3), 3.65 (dd, 1H, *J*_5,6b_ = 5.7, *J*_6a,6b_ = 13.2, H-6b), 3.88 (dd, 1H, *J*_5,6a_ = 2.6, H-6a), 5.09 (d, 1H, *J*_1,2_ = 10.1, H-1). ^13^C NMR (D_2_O): δ 25.9 (C-8), 40.8 (C-9), 53.5 (*Me*SO_2_), 61.9, 70.9, 73.2, 79.0, 81.7, 83.9 (C-1-C-6), 163.9 (*C*=N). HR-ESI-MS *m*/*z* calcd for C_10_H_18_NO_11_S_3_ [M−H]^−^ 424.0042, found 424.0031.

*3-(Methylsulfonyl)propyl glucosinolate* *(***4b***). Glucocheirolin* [15592-36-6].

White amorphous powder (0.093 g, 85% yield), [α]_D_ -20 (*c* = 0.8, H_2_O). ^1^H NMR (D_2_O): δ 1.95–2.06 (m, 2H, H-9), 2.70 (t, 2H, *J* = 7.5, H-8), 2.99 (s, 3H, *Me*SO_2_), 3.21 (t, 2H, *J* = 7.3, H-10), 3.45–3.56 (m, 2H, H-2, H-4), 3.58–3.67 (m, 2H, H-5, H-3), 3.72 (dd, 1H, *J*_5,6b_ = 5.8, *J*_6a,6b_ = 13.1, H-6b), 3.93 (dd, 1H, *J*_5,6a_ = 2.7, H-6a), 5.09 (d, 1H, *J*_1,2_ = 10.2, H-1). ^13^C NMR (D_2_O): δ 21.9, 31.1, 40.7 (C-8-C-10), 54.2 (*Me*SO_2_), 61.8, 70.8, 73.3, 78.9, 81.5, 83.5 (C-1-C-6), 164.5 (*C*=N). HR-ESI-MS *m*/*z* calcd for C_11_H_20_NO_11_S_3_ [M−H]^−^ 438.0198, found 438.0184.

*4-(Methylsulfonyl)butyl glucosinolate* *(***4c***). Glucoerysolin* [22149-26-4].

White amorphous powder (0.098 g, 87% yield), [α]D -22 (c = 1.1, H_2_O). ^1^H NMR (D_2_O): δ 1.48–1.81 (m, 4H, H-9, H-10), 2.56 (t, 2H, *J* = 7.4, H-8), 2.96 (s, 3H, *Me*SO_2_), 3.16 (t, 2H, *J* = 7.3, H-11), 3.40–3.54 (m, 2H, H-2, H-4), 3.56–3.66 (m, 2H, H-5, H-3), 3.76 (dd, 1H, *J*_5,6b_ = 5.8, *J*_6a,6b_ = 13.1, H-6b), 3.91 (dd, 1H, *J*_5,6a_ = 2.6, H-6a), 5.09 (d, 1H, *J*_1,2_ = 10.0, H-1). ^13^C NMR (D_2_O): δ 22.6, 26.4, 32.2, 40.9 (C-8-C-11), 54.9 (*Me*SO_2_), 61.5, 70.9, 73.2, 79.2, 81.7, 84.1 (C-1-C-6), 165.0 (C=N). HR-ESI-MS *m*/*z* calcd for C_12_H_22_NO_11_S_3_ [M−H]^−^ 452.0355, found 452.0343.

*5-(Methylsulfonyl)pentyl glucosinolate* *(***4d***)* [666235-38-7].

White amorphous powder (0.105 g, 90% yield), [α]_D_ -21 (*c* = 0.8, H_2_O). ^1^H NMR (D_2_O): δ 1.52–1.64 (m, 2H, H-10), 1.69–1.94 (m, 4H, H-9, H-11), 2.68 (t, 2H, *J* = 7.7, H-8), 2.98 (s, 3H, *Me*SO_2_), 3.18 (t, 2H, *J* = 7.4, H-12), 3.44–3.52 (m, 2H, H-2, H-4), 3.55–3.68 (m, 2H, H-5, H-3), 3.74 (dd, 1H, *J*_5,6b_ = 5.8, *J*_6a,6b_ = 13.2, H-6b), 3.89 (dd, 1H, *J*_5,6a_ = 2.7, H-6a), 5.09 (d, 1H, *J*_1,2_ = 10.2, H-1). ^13^C NMR (D_2_O): δ 22.8, 26.9, 28.0, 32.4, 40.9 (C-8-C-12), 54.6 (*Me*SO_2_), 62.4, 71.5, 73.2, 78.9, 82.2, 83.6 (C-1-C-6), 165.1 (*C*=N). HR-ESI-MS *m*/*z* calcd for C_13_H_24_NO_11_S_3_ [M−H]^−^ 466.0511, found 466.0501.

*7-(Methylsulfonyl)heptyl glucosinolate* *(***4e***)* [862388-51-0].

White amorphous powder (0.110 g, 90% yield), [α]_D_ -18 (*c* = 0.8, H_2_O). ^1^H NMR (D_2_O): δ 1.24–1.42 (m, 6H, H-10-H-12), 1.43–1.64 (m, 4H, H-9, H-13), 2.48–2.61 (m, 2H, H-8), 2.96 (s, 3H, *Me*SO_2_), 3.18 (t, 2H, *J* = 7.8, H-14), 3.44–3.52 (m, 2H, H-2, H-4), 3.58–3.65 (m, 2H, H-5, H-3), 3.71 (dd, 1H, *J*_5,6b_ = 5.8, *J*_6a,6b_ = 13.1, H-6b), 3.89 (dd, 1H, *J*_5,6a_ = 2.7, H-6a), 5.03 (d, 1H, *J*_1,2_ = 10.0, H-1). ^13^C NMR (D_2_O): δ 21.8, 26.4, 27.8, 28.2, 28.3, 32.4, 40.5 (C-8-C-14), 54.5 (*Me*SO_2_), 61.8, 70.7, 73.0, 79.3, 81.6, 83.3 (C-1-C-6), 164.0 (*C*=N). HR-ESI-MS *m*/*z* calcd for C_15_H_28_NO_11_S_3_ [M−H]^−^ 494.0823, found 494.0815.

*9-(Methylsulfonyl)nonyl glucosinolate* *(***4f***)* [192580-85-1].

White amorphous powder (0.121 g, 94% yield), [α]_D_ -21 (*c* = 0.9, H_2_O). ^1^H NMR (D_2_O): δ 1.22–1.40 (m, 10H, H-10-H-14), 1.42–1.65 (m, 4H, H9, H15), 2.51–2.66 (m, 2H, H-8), 2.98 (s, 3H, *Me*SO_2_), 3.16 (t, 2H, *J* = 7.4, H-16), 3.41–3.49 (m, 2H, H-2, H-4), 3.55–3.67 (m, 2H, H-5, H-3), 3.75 (dd, 1H, *J*_5,6b_ = 5.8, *J*_6a,6b_ = 13.1, H-6b), 3.92 (dd, 1H, *J*_5,6a_ = 2.6, H-6a), 5.04 (d, 1H, *J*_1,2_ = 10.1, H-1). ^13^C NMR (D_2_O): δ 22.5, 26.6, 27.8, 29.5, 29.6br, 30.0, 32.9, 40.1 (C-8-C-16), 54.2 (*Me*SO_2_), 62.2, 71.2, 73.4, 79.0, 81.5, 83.8 (C-1-C-6), 165.2 (*C*=N) [38]. HR-ESI-MS *m*/*z* calcd for C_17_H_32_NO_11_S_3_ [M−H]^−^ 522.1136, found 522.1122.

*11-(Methylsulfonyl)undecyl glucosinolate* *(***4g***).*

White amorphous powder (0.129 g, 95% yield), [α]_D_ -20 (*c* = 0.8, H_2_O). ^1^H NMR (D_2_O): δ 1.22–1.44 (m, 14H, H-10-H-16), 1.45–1.69 (m, 4H, H-9, H-17), 2.50–2.65 (m, 2H, H-8), 2.98 (s, 3H, *Me*SO_2_), 3.15 (t, 2H, *J* = 7.6, H-18), 3.43–3.50 (m, 2H, H-2, H-4), 3.52–3.63 (m, 2H, H-5, H-3), 3.73 (dd, 1H, *J*_5,6b_ = 5.8, *J*_6a,6b_ = 13.2, H-6b), 3.91 (dd, 1H, *J*_5,6a_ = 2.7, H-6a), 5.09 (d, 1H, *J*_1,2_ = 10.1, H-1). ^13^C NMR (D_2_O): δ 22.6, 27.6, 28.9, 29.0br, 29.8, 30.0, 30.4br, 33.6, 40.6 (C-8-C-18), 54.6 (*Me*SO_2_), 61.7, 70.4, 73.2, 78.6, 82.4, 83.6 (C-1-C-6), 165.2 (*C*=N). HR-ESI-MS *m*/*z* calcd for C_19_H_36_NO_11_S_3_ [M−H]^−^ 550.1449, found 550.1442.

#### 3.2.3. Pathway 2

##### Selective Oxidation of ω-Methylsulfanylnitroalkanes **10** to ω-Sulfoxides **11**

ω-Methylsulfanylnitroalkanes 10a–g were obtained as previously described in [29]. To a stirred solution of ω-methylsulfanylnitroalkane, **10** (12 mmol) in methanol (70 mL) cooled at 0 °C, an ice-cold solution of NaIO_4_ (25 mmol) in water (35 mL) was added dropwise. After stirring at r.t. for 1.5–3 h, the mixture was filtered, and the clear solution was diluted with water (100 mL) and then repeatedly extracted with chloroform (4 × 50 mL). The combined organic phases were dried over Na_2_SO_4_, filtered, and concentrated in vacuo. The residue was purified by flash column chromatography (dichloromethane containing 3–25% *v*/*v* methanol) to produce sulfoxides **11a**–**g** as viscous oils or waxy solids.

*(1-Methylsulfinyl)-3-nitropropane* *(***11a***).*

Yellowish oil (1.09 g, 60% yield). ^1^H NMR (CDCl_3_): δ 2.34–2.43 (m, 2H, H-2), 2.52 (s, 3H, *Me*SO), 2.69 (t, 2H, *J*_1,2_ = 7.3, H-1), 4.50 (t, 2H, *J*_3,2_ = 6.8, H-3). ^13^C NMR (CDCl_3_): δ 20.7 (C-2), 38.2 (*Me*SO), 49.9 (C-1), 73.5 (C-3). C_4_H_9_NO_3_S MS IS *m*/*z* = 152.2 [M+H]^+^.

*(1-Methylsulfinyl)-4-nitrobutane* *(***11b***).*

Yellowish oil (1.25 g, 63% yield). ^1^H NMR (CDCl_3_): δ 1.76–1.84 (m, 2H, H-2), 2.04–2.13 (m, 2H, H-3), 2.49 (s, 3H, *Me*SO), 2.65 (t, 2H, *J*_1,2_ = 7.3, H-1), 4.36 (t, 2H, *J*_4,3_ = 6.8, H-4). ^13^C NMR (CDCl_3_): δ 23.3 (C-2), 25.8 (C-3), 38.2 (*Me*SO), 52.8 (C-1), 74.6 (C-4). C_5_H_11_NO_3_S MS IS *m*/*z* = 166.2 [M+H]^+^.

*(1-Methylsulfinyl)-5-nitropentane* *(***11c***).*

Yellowish oil (1.76 g, 82% yield). ^1^H NMR (CDCl_3_): δ 1.39–1.53 (m, 2H, H-3), 1.69–1.80 (m, 2H, H-2), 1.92–2.01 (m, 2H, H-4), 2.48 (s, 3H, *Me*SO), 2.56–2.65 (m, 2H, H-1), 4.32 (t, 2H, *J*_5,4_ = 6.8, H-5). ^13^C NMR (CDCl_3_): δ 21.6 (C-2), 25.1 (C-3), 26.5 (C-4), 38.3 (*Me*SO), 53.6 (C-1), 74.9 (C-5). C_6_H_13_NO_3_S MS IS *m*/*z* = 180.2 [M+H]^+^.

*(1-Methylsulfinyl)-6-nitrohexane* *(***11d***).*

Yellowish wax (2.06 g, 89% yield). ^1^H NMR (CDCl_3_): δ 1.26–1.46 (m, 4H, H-3, H-4), 1.61–1.71 (m, 2H, H-2), 1.85–1.94 (m, 2H, H-5), 2.44 (s, 3H, *Me*SO), 2.52–2.60 (m, 2H, H-1), 4.27 (t, 2H, *J*_6,5_ = 6.8, H-6). ^13^C NMR (CDCl_3_): δ 21.8 (C-2), 25.4 (C-3), 26.5 (C-4), 27.5 (C-5), 38.1 (*Me*SO), 53.8 (C-1), 75.1 (C-6). C_7_H_15_NO_3_S MS IS *m*/*z* = 194.3 [M+H]^+^.

*(1-Methylsulfinyl)-8-nitrooctane* *(***11e***).*

Yellowish wax (2.34 g, 88% yield). ^1^H NMR (CDCl_3_): δ 1.22–1.41 (m, 8H, H-3-H-6), 1.63–1.70 (m, 2H, H-2), 1.85–1.95 (m, 2H, H-7), 2.48 (s, 3H, *Me*SO), 2.55–2.67 (m, 2H, H-1), 4.30 (t, 2H, *J*_8,7_ = 7.0, H-8). ^13^C NMR (CDCl_3_): δ 22.1 (C-2), 27.0 (C-7), 25.8, 28.2, 28.5 (C-3-C-6), 38.3 (*Me*SO), 54.3 (C-1), 75.4 (C-8). C_9_H_19_NO_3_S MS IS *m*/*z* = 222.3 [M+H]^+^.

*(1-Methylsulfinyl)-10-nitrodecane* *(***11f***).*

Yellowish wax (2.81 g, 94% yield). ^1^H NMR (CDCl_3_): δ 1.25–1.44 (m, 12H, H-3-H-8), 1.66–1.74 (m, 2H, H-2), 1.90–1.99 (m, 2H, H-9), 2.51 (s, 3H, *Me*SO), 2.58–2.71 (m, 2H, H-1), 4.33 (t, 2H, *J*_10,9_ = 7.0, H-10). ^13^C NMR (CDCl_3_): δ 22.3 (C-2), 27.1 (C-9), 25.9, 28.5, 28.9, 29.2 (C-3-C-8), 38.4 (*Me*SO), 54.5 (C-1), 75.6 (C-10). C_11_H_23_NO_3_S MS IS *m*/*z* = 250.4 [M+H]^+^.

*(1-Methylsulfinyl)-12-nitrododecane* *(***11g***).*

Yellowish wax (3.06 g, 92% yield). ^1^H NMR (CDCl_3_): δ 1.19–1.43 (m, 16H, H-3-H-10), 1.63–1.73 (m, 2H, H-2), 1.88–1.98 (m, 2H, H-11), 2.49 (s, 3H, *Me*SO), 2.56–2.68 (m, 2H, H-1), 4.31 (t, 2H, *J*_12,11_ = 7.1, H-12). ^13^C NMR (CDCl_3_): δ 22.3 (C-2), 27.1 (C-11), 25.9, 28.5, 28.9, 29.0, 29.1 (C-3-C-10), 38.3 (*Me*SO), 54.5 (C-1), 75.5 (C-12). C_13_H_27_NO_3_S MS IS *m*/*z* = 278.4 [M+H]^+^.

##### Selective Oxidation of ω-Methylsulfanylnitroalkanes **10** to ω-Sulfones **12**

To a stirred solution of ω-methylsulfanylnitroalkane, **10** (10 mmol) in methanol (80 mL) cooled at 0 °C, a solution of oxone (30 mmol) in water (45 mL) was added dropwise. After stirring at r.t. for 2–3 h, the white suspension was filtered, and the clear solution was concentrated (below 30 °C) in vacuo, then repeatedly extracted with chloroform (4 × 50 mL). The combined organic phases were dried over Na_2_SO_4_, filtered, and concentrated in vacuo. The residue was purified by flash column chromatography (dichloromethane containing 3–25% *v*/*v* methanol) to produce sulfones **12a**–**g** as viscous oils or waxy solids.

*(1-Methylsulfonyl)-3-nitropropane* *(***12a***).*

Yellowish oil (1.20 g, 72% yield). ^1^H NMR (CDCl_3_): δ 2.46–2.56 (m, 2H, H-2), 2.94 (s, 3H, *Me*SO_2_), 3.16 (t, 2H, *J*_1,2_ = 7.3, H-1), 4.59 (t, 2H, *J*_3,2_ = 6.4, H-3). ^13^C NMR (CDCl_3_): δ 19.7 (C-2), 40.8 (*Me*SO_2_), 50.8 (C-1), 72.9 (C-3). C_4_H_9_NO_4_S MS IS *m*/*z* = 168.2 [M+H]^+^.

*(1-Methylsulfonyl)-4-nitrobutane* *(***12b***).*

Yellowish oil (1.59 g, 88% yield). ^1^H NMR (CDCl_3_): δ 1.82–1.93 (m, 2H, H-2), 2.06–2.16 (m, 2H, H-3), 2.86 (s, 3H, *Me*SO_2_), 3.03 (t, 2H, *J*_1,2_ = 7.5, H-1), 4.40 (t, 2H, *J*_4,3_ = 6.8, H-4). ^13^C NMR (CDCl_3_): δ 19.0 (C-2), 25.4 (C-3), 40.4 (*Me*SO_2_), 53.1 (C-1), 74.4 (C-4). C_5_H_11_NO_4_S MS IS *m*/*z* = 182.2 [M+H]^+^.

*(1-Methylsulfonyl)-5-nitropentane* *(***12c***).*

Yellowish wax (1.83 g, 94% yield). ^1^H NMR (CDCl_3_): δ 1.48–1.58 (m, 2H, H-3), 1.82–1.92 (m, 2H, H-2), 1.97–2.07 (m, 2H, H-4), 2.88 (s, 3H, *Me*SO_2_), 3.00 (ft, 2H, *J*_1,2_ = 8.0, H-1), 4.38 (t, 2H, *J*_5,4_ = 6.8, H-5). ^13^C NMR (CDCl_3_): δ 21.4 (C-2), 24.9 (C-3), 26.5 (C-4), 40.5 (*Me*SO_2_), 53.9 (C-1), 74.9 (C-4). C_6_H_13_NO_4_S MS IS *m*/*z* = 196.2 [M+H]^+^.

*(1-Methylsulfonyl)-6-nitrohexane* *(***12d***).*

Yellowish wax (1.92 g, 92% yield). ^1^H NMR (CDCl_3_): δ 1.37–1.52 (m, 4H, H-3, H-4), 1.78–1.86 (m, 2H, H-2), 1.94–2.04 (m, 2H, H-5), 2.87 (s, 3H, *Me*SO_2_), 2.98 (ft, 2H, *J*_1,2_ = 8.0, H-1), 4.36 (t, 2H, *J*_6,5_ = 6.8, H-6). ^13^C NMR (CDCl_3_): δ 21.8 (C-2), 25.5 (C-3), 27.3 (C-4), 40.4 (*Me*SO_2_), 54.2 (C-1), 75.3 (C-6). C_7_H_15_NO_4_S MS IS *m*/*z* = 210.3 [M+H]^+^.

*(1-Methylsulfonyl)-8-nitrooctane* *(***12e***).*

Yellowish wax (2.28 g, 96% yield). ^1^H NMR (CDCl_3_): δ 1.36–1.50 (m, 8H, H-3-H-6), 1.80–1.89 (m, 2H, H-2), 1.95–2.04 (m, 2H, H-7), 2.89 (s, 3H, *Me*SO_2_), 2.99 (ft, 2H, *J*_1,2_ = 8.0, H-1), 4.37 (t, 2H, *J*_8,7_ = 6.8, H-8). ^13^C NMR (CDCl_3_): δ 22.0 (C-2), 27.0 (C-7), 25.8, 27.9, 28.2, 28.4 (C-3-C-6), 40.3 (*Me*SO_2_), 54.5 (C-1), 75.5 (C-8). C_9_H_19_NO_4_S MS IS *m*/*z* = 238.3 [M+H]^+^.

*(1-Methylsulfonyl)-10-nitrodecane* *(***12f***).*

Yellowish wax (2.60 g, 98% yield). ^1^H NMR (CDCl_3_): δ 1.25–1.46 (m, 12H, H-3-H-8), 1.78–1.89 (m, 2H, H-2), 1.95–2.02 (m, 2H, H-9), 2.89 (s, 3H, *Me*SO_2_), 2.99 (ft, 2H, *J*_1,2_ = 8.0, H-1), 4.37 (t, 2H, *J*_10,9_ = 6.8, H-10). ^13^C NMR (CDCl_3_): δ 22.2 (C-2), 27.1 (C-9), 25.9, 28.1, 28.5, 28.7, 28.8 (C-3-C-8), 40.3 (*Me*SO_2_), 54.6 (C-1), 75.6 (C-10). C_11_H_23_NO_4_S MS IS *m*/*z* = 266.4 [M+H]^+^.

*(1-Methylsulfonyl)-12-nitrododecane* *(***12g***).*

Yellowish wax (2.79 g, 95% yield). ^1^H NMR (CDCl_3_): δ 1.26–1.46 (m, 16H, H-3-H-10), 1.79–1.89 (m, 2H, H-2), 1.94–2.02 (m, 2H, H-11), 2.89 (s, 3H, *Me*SO_2_), 2.99 (ft, 2H, *J*_1,2_ = 8.0, H-1), 4.37 (t, 2H, *J*_12,11_ = 6.8, H-12). ^13^C NMR (CDCl_3_): δ 22.2 (C-2), 28.2 (C-11), 26.0, 28.2, 28.6, 28.8, 29.0, 29.1, 29.2 (C-3-C-10), 40.3 (*Me*SO_2_), 54.7 (C-1), 75.7 (C-12). C_13_H_27_NO_4_S MS IS *m*/*z* = 294.4 [M+H]^+^.

##### General Procedure for Nitronate Chlorination and Coupling with the Thioglucose Unit

The nitro derivatives **11** or **12** were transformed into the target thiohydroximates **6** or **7** through a three-step strategy involving a. nitronate formation, b. conversion of nitronate into hydroximoyl chloride and final c. coupling with 2,3,4,6-tetra-*O*-acetyl-1-thio-*β*-d-glucopyranose, according to a well-established procedure previously reported in full detail [29]. The glycosyl thiohydroximates **6** or **7** were obtained as amorphous solids. The yield range was 30–54% for the ω-methylsulfinylalkyl thiohydroximates **6** and 35–55% for their ω-methylsulfonylalkyl counterparts **7**. The yield for each compound is presented in Table 2. **6a**–**g** and **7a**–**g** were described above in Section 3.2.1.

## 4. Conclusions

We developed two general pathways for the synthesis of biologically critical ω-methylsulfinylalkyl GSLs and their ω-methylsulfonylalkyl counterparts. A first approach based on selective sulfur oxidation of a range of ω-methylsulfanyl analogs proved practicable, delivering the expected thiofunctionalized GSLs in moderate overall yields. An alternative method involving tailor-made ω-methylsulfinyl- and ω-methylsulfonyl nitroalkanes was also developed. Using a well-established protocol, those nitroalkanes precursors were converted into transient nitrile oxides to be coupled with *O*-protected 1-thio-β-d-glucopyranose, producing the intermediate thiohydroximates in a slightly higher overall yield, compared to the first approach. In conclusion, both synthetic pathways make available attractive GSLs with a thiofunctionalyzed aglycon chain for biological studies. Noteworthy, the biological activity of intact GSLs has been long overlooked in favor of a large number of studies on their renowned hydrolysis product ITCs. Emerging evidence suggests that GSLs are endowed with biological properties mediated by H_2_S-releasing or -inducing properties that require further elucidation.

## Figures and Tables

**Table 1:** Pathway 1—synthesis of protected glucosyl thiohydroximate **6a**–**g** and **7a**–**g** by selective oxidation of ω-methylsulfanylalkyl precursors **5a**–**g**.

Chain Size (n)	cpd	Yield (%) [29]	cpd	Yield (%)	Overall Yield (%)	cpd	Yield (%)	Overall Yield (%)
2	**5a**	32	**6a**	42	13	**7a**	49	16
3	**5b**	34	**6b**	49	17	**7b**	54	18
4	**5c**	40	**6c**	53	21	**7c**	56	22
5	**5d**	48	**6d**	51	24	**7d**	60	29
7	**5e**	47	**6e**	60	28	**7e**	68	32
9	**5f**	51	**6f**	68	35	**7f**	65	33
11	**5g**	56	**6g**	65	36	**7g**	70	39

**Table 2:** Pathway 2—synthesis of protected glucosyl thiohydroximate **6a**–**g** and **7a**–**g** by nitronate chlorination and coupling with the thioglucose unit approach.

Chain Size (n)	cpd	Yield (%)	cpd	Yield (%)	Overall Yield (%)	cpd	Yield (%)	cpd	Yield (%)	Overall Yield (%)
2	**11a**	60	**6a**	30	18	**12a**	72	**7a**	35	25
3	**11b**	63	**6b**	31	20	**12b**	88	**7b**	36	32
4	**11c**	82	**6c**	38	31	**12c**	94	**7c**	40	38
5	**11d**	89	**6d**	46	41	**12d**	92	**7d**	45	41
7	**11e**	88	**6e**	45	40	**12e**	96	**7e**	48	46
9	**11f**	94	**6f**	54	51	**12f**	98	**7f**	52	51
11	**11g**	92	**6g**	51	47	**12g**	95	**7g**	55	52

## Data Availability

Data are contained within the article.
